# The Effect of X-ray Irradiation on the Fitness and Field Adaptability of the Codling Moth: An Orchard Study in Northeast China

**DOI:** 10.3390/insects14070615

**Published:** 2023-07-07

**Authors:** Jinghan Zhang, Shengwang Huang, Shici Zhao, Xingya Wang, Xianming Yang, Huiyuan Zhao, Ping Gao, Yuting Li, Xueqing Yang

**Affiliations:** 1 College of Plant Protection, Shenyang Agricultural University, Shenyang 110866, China; yutingli2017@syau.edu.cn (Y.L.); 2 Key Laboratory of Economical and Applied Entomology of Liaoning Province, Shenyang 110866, China; 3 State Key Laboratory for Biology of Plant Diseases and Insect Pests, Institute of Plant Protection, Chinese Academy of Agricultural Sciences, Beijing 100193, China; zqbxming@163.com; 4 Hebi Jiaduoke Industry and Trade Co., Ltd., Hebi 458030, China

**Keywords:** *Cydia pomonella*, physical prevention and control, sterile insect technology, X-ray irradiation, mating competition

## Abstract

**Simple Summary:**

The codling moth, *Cydia pomonella* (L.), is an invasive agricultural pest species of pome fruits and walnuts that has developed resistance to many insecticides. Alternative eco-friendly approaches are warranted to reduce the dependence on insecticides for the sustainable management of *C. pomonella*. The sterile insect technique (SIT) is an autocidal strategy of control that is being trialed in experiments against various pests and that could reduce the risk of the development of insecticide resistance. In our previous study, we found that 366 Gy X-ray irradiation can effectively make the *C. pomonella* male sterile. In this study, we report on the investigation of the effect of X-ray irradiation on the fitness and adaptability of sterile insects, as well as the first pilot release of sterile male adults of *C. pomonella* in orchards in China. Results show that 366 Gy of X-ray irradiation significantly shortened the lifespan of the sterile male moths, reduced the mating competitiveness of males, and resulted in males being sterile in the orchards.

**Abstract:**

The codling moth, *Cydia pomonella* (L.), is an invasive agricultural pest of pome fruits and walnuts in China that threatens the apple industry in the Loess Plateau and Bohai Bay; it has developed resistance to many insecticides. Sterile insect technique (SIT) combined with area-wide integrated pest management (AW-IPM) can reduce the risk of resistance to insecticides and effectively control some insect pest species. Our previous laboratory experiment found that irradiation with 366 Gy of X-ray caused the males of the codling moth to become sterile. However, the sterility and adaptability of males after being irradiated with 366 Gy X-ray in the field are still unclear. In this study, we investigated the effect of X-ray irradiation on the fitness of male adults that emerged from pupae irradiated with 366 Gy to explore their adaptability and mating competitiveness, and to examine the effect of releasing sterile male insects in orchards in northeast China on the fruit infestation rate of the Nanguo pear. The results showed that 366 Gy of X-ray irradiation significantly reduced the mating competitiveness of males and the hatching rate of the eggs laid by females pairing with sterile males. Meanwhile, the lifespan of the sterile male moths was significantly shorter than that of the normal ones in the field. A pilot test showed that the release twice of sterile male moths in the orchards had no significant effect on the fruit infestation rate. Our field experiments provide a scientific basis for the further optimization of the SIT technology program for controlling *C. pomonella*.

## 1. Introduction

The codling moth, *Cydia pomonella* (L.), is a devastating pest in pome fruits and walnuts worldwide [1,2]. This pest is also an invasive agricultural pest species in China [3]. The larvae of *C. pomonella* bore into the center of fruits to feed on the pulp and seeds, leaving frass on the surface and causing the abscission of fruits [4]. Generally, one larva may damage 3–4 fruits because of the characteristic of fruit switching [5]. As a result, it has been listed in the list of invasive alien species under key management issued by six departments, including the Ministry of Agriculture and Rural Affairs, the Ministry of Natural Resources, the Ministry of Ecology and Environment, the Ministry of Housing and Urban–Rural Development, the General Administration of Customs, and the State Forestry and Grass Administration of China (PRC). The codling moth originated from Eurasia and was first reported in the Xinjiang Uygur Autonomous Region of China in 1957 [6]. Since the insect’s invasion of China, the distribution of *C. pomonella* has extended to 195 counties in nine provinces as of 2021, including Xinjiang, Gansu, Ningxia, Inner Mongolia, Heilongjiang, Jilin, Liaoning, Hebei, and Tianjin, with the occurrence area reaching 40,000 hm^2^, which causes annual economic losses up to USD 605 million [7,8,9].

Currently, *C. pomonella* is controlled by agricultural, physical, biological, and chemical measures [10]. Generally, the egg and initial larval stages of *C. pomonella* are the key periods for chemical control. However, with the characteristics of generations overlapping and the short time that neonate larvae stay outside of the fruits after egg hatching, this pest is difficult to control using chemical insecticides. Moreover, the extensive use of insecticides has resulted in the development of *C. pomonella* resistance to a range of chemical insecticides [11,12], particularly organophosphates [13] and pyrethroids [14], and it may also cause biodiversity reduction and insecticide residues on fruits [15]. Therefore, alternative eco-friendly approaches are warranted to reduce the dependence on insecticides for the sustainable management of *C. pomonella*.

Sterile insect technique (SIT) is an eco-friendly species-specific control method used in pest management, which is usually used as a part of the integrated pest management in large areas to control pests. SIT is not a stand-alone technique requiring the prior suppression of pest populations in most cases [16,17]. Exposure to ionizing radiation such as X-rays, electron beams, or γ-rays is a common treatment method for yielding sterile insects using SIT. After a large number of sterile insects are released into the field, they can mate with virgin females to produce infertile offspring and reduce the population density [18]. Unlike other control methods, releasing sterile males does not directly impact the pest population but reduces its offspring. Therefore, the number of pests can be reduced by the continuous release of sterile insects in the field to inhibit population expansion [19,20].

SIT has been successfully applied as a part of the AW-IPM program for the eradication of some pest species, such as *Cochliomyia hominivorax* (Coquerel) in America [21] and *C. pomonella* [22,23] in Canada. Many investigations have focused on producing sterile insects and on the competitiveness of irradiated insects, for example, *Ephestia elutella* [24], *Aedes aegypti* and *A. albopictus* [25], and *C. pomonella* [26]. However, radioactive materials are hazardous, and their usage is becoming increasingly limited. There is an urgent need to find alternative sources of radiation. Studies have shown that X-ray irradiation is a safer alternative to gamma radiation; it is easily obtained and operated, and it can fulfill SIT programs’ requirements [27,28]. Jiang [29] found that the sterility ratio of *Spodoptera litura* was 84% at the most appropriate irradiation dose (71.26 Gy) and that it could achieve 91% sterility in an indoor mating competition experiment when the release ratio of irradiated males (75 Gy) to non-irradiated males reached 12.6:1.

Our previous study has documented that the 8-day-old male pupa was the most suitable for X-ray irradiation [28]. Also, the proportions of sub-sterile (20.93%) and completely sterile (100%) males were determined by using 183 Gy and 366 Gy, respectively. Laboratory experiments showed that the mating competitiveness of male moths decreased at 366 Gy of X-ray irradiation but still had potential in the SIT program for controlling the codling moth [28]. Therefore, the present study aims to explore whether the 366 Gy of X-ray-irradiated males are sterile in the field. The effects of X-ray irradiation on the fitness and field adaptability of *C. pomonella* in an orchard from northeast China were assessed.

## 2. Materials and Methods

### 2.1. Insects

A *C. pomonella* strain [30] was reared on an artificial diet under laboratory conditions of 26 ± 1 °C, 60 ± 5% relative humidity (RH), and a 16:8 h (L:D) photoperiod for more than 50 generations without exposure to any ionizing radiation.

### 2.2. Irradiation

A new X-ray irradiator (JYK-001 type), independently developed by Hebi Jiaduoke Industry and Trade Co., Ltd., Hebi, China, was used in this study. The equipment used in this test is the same as in the previous test [28]. The irradiation dose used in this study is the average dose, and the dose rate was measured using a Radcal Accu-Dose+ digitizer with a 10 × 6–0.6 CT ion chamber. The 8-day-old male pupae were irradiated with 366 Gy X-ray at a dose rate of 12.7083 mGy/s, and their fitness was assessed in a Nanguo pear orchard.

### 2.3. Orchard Cage Experiments

#### 2.3.1. Effect of X-ray Irradiation on the Lifespan, Fecundity, and Fertility of Male Adults of *C. pomonella*

**Figure 1:**
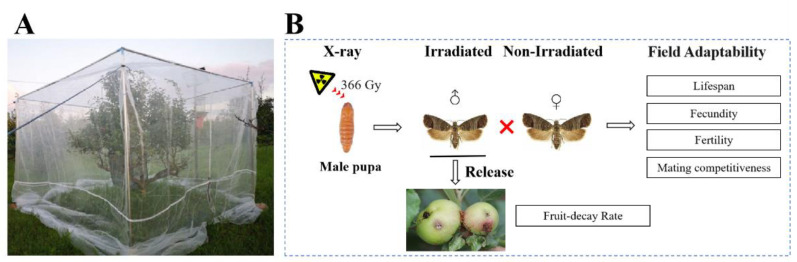
Experimental design for the effect of X-ray irradiation on the fitness and field adaptability of the codling moth in a Nanguo pear orchard. A tree was installed with a stand and a high-density (40 mesh) insect net, and a small insect feeding net (60 cm × 60 cm × 90 cm) was fixed on the tree with a waxed paper-lined cage (25 × 12 × 15 cm) placed inside (**A**). After irradiating the 8-day-old male pupae with 366 Gy of X-ray, the moths that emerged were paired with non-irradiated females. The lifespan of the male adults that emerged, the fecundity and fertility of the females that mated with males irradiated with 366 Gy X-ray irradiation, and the non-irradiated (control), as well as the mating competitiveness of males, were recorded. Furthermore, the effect of the pilot release of sterile male adults of *C. pomonella* on the fruit infestation rate of the Nanguo pear was also determined (**B**).

A tree was installed with a stand and a high-density (40 mesh) insect net (3.5 m × 3.5 m × 3.5 m) (Figure 1) in the Nanguo pear orchard in Zhangwu County (42°28′42″ N, 122°7′5″ E), Liaoning Province, China. A small insect feeding net (60 cm × 60 cm × 90 cm) was fixed on the pear tree inside the high-density insect net, with a waxed paper-lined cage (25 × 12 × 15 cm) placed inside. The 8-day-old male pupae (8 days being the most suitable age for irradiation) were irradiated with 366 Gy of X-ray as described previously [28]. After the emergence of the moths, the irradiated males and non-irradiated females were randomly paired at a sex ratio of 1:1 in the waxed paper-lined cage. The moths were provided with a 10% honey solution on absorbent cotton. Three groups, each with 8 pairs, were formed, with the non-irradiated males instead of the irradiated males used as the controls. The cage was placed in the field, and the survival of each male adult was observed every day, and the lifespan of male adults was recorded. The emerging moths were paired with non-irradiated female adults to allow them to lay eggs on the waxed papers. The waxed papers were collected, and the number of eggs laid and hatched was recorded daily. The mate capsule of the dead female adults was dissected under a stereoscope with 1 × objective (TS-63X, Shanghai Shangguang New Optical Technology Co., Ltd., Shanghai, China) to check whether there was a spermatophore, in order to calculate the single female oviposition. During the field experiment, the weather conditions were also recorded every day.

#### 2.3.2. Effect of X-ray Irradiation on the Mating Competitiveness of Male *C. pomonella*

Irradiated males (IM), non-irradiated males (NM), and non-irradiated females (NF) were introduced into waxed paper spawning cages (25 cm × 12 cm × 15 cm) at the ratio of 0:1:1, 1:0:1, and 1:1:1, respectively, for mating. Each mating ratio combination was repeated three times, and each contained 8 insects. For example, IM:NM:NF = 0:1:1 means that 0 irradiated males: 8 non-irradiated males: 8 non-irradiated females were introduced into a cage for mating. Then, the total number of eggs laid and hatched in each group was recorded. According to the method of [31], the competitive mating index (C) was calculated using the following formula:

$$E=\frac{N(Ha)+S(Hs)}{N+S}$$

$$C=\frac{Ha-Ee}{He-Hs}\;÷\;\frac{S}{N}$$

*N* is the number of NM. *S* is the number of IM. Ha is the egg-hatching rate (hatched/total eggs) of NM paired with NF, whereas *Hs* is the egg-hatching rate of IM paired with NF. *Ee* represents the observed hatching rate of mixed male adults with different *S*/*N* ratios paired with normal female adults. E represents the expected hatching rate of mixed male adults with different *S*/*N* ratios paired with normal female adults, respectively.

#### 2.3.3. Effect of Pilot Release of Sterile Male Adults of *C. pomonella* on Fruit Infestation Rate of Nanguo Pear

A Nanguo pear orchard with a size of 500 acres was used in this study. Of this area, 25 acres of the orchard were used for the pilot release of sterile male adults of *C. pomonella*, and another 25 acres of the orchard were used for control. To ensure that the sterile males of the experimental group released in the orchard center and the wild males in the control group would not interfere with each other, a blank area of land of several hundred meters between the pilot release area and the control orchard was set. On the day before the release of the sterile males, 25 triangle traps (Beijing Zhongjie Sifang Biotechnology Co., Ltd., Beijing, China), each with a lure core containing female pheromones placed above a sticky board, were randomly arranged on the trees in the orchard. A total of 12 moths were trapped in these traps for 1 week, which represents the initial number of *C. pomonella* in the orchard. A total of 20 fruit trees were randomly selected, and a five-point sampling method was used to investigate the fruit infestation rate of the trees in the four corners (east, south, west, and north). The fruits with hanging insect droppings and wormholes were counted as rotten fruits, and this number was used to calculate the fruit infestation rate of the Nanguo pear in the control and the experimental orchards, respectively. When being released, irradiated males placed in Petri dishes were released at a height of about 1.5 m above the ground in the center of the orchard. Based on our previous study, the estimated mating competitiveness of sterile males was 0.0088 (0.01), which means that about 113 sterile males were as competitive as 1 normal male. Thus, we released twice (22 July 2022, and 2 September 2022), each time with 1250 sterile males, respectively, because the female codling moth had mated more than once based on our observation in the laboratory. Sterile moths were not released in the control orchard. The fruit infestation rate of each group was checked every 10 days post-release to evaluate whether there was a control effect between the two orchards.

### 2.4. Data Analysis

Significant differences in the mean value of the mortality and the fruit infestation rate were subjected to two-way ANOVA (*p* < 0.05), and the number of eggs laid (fecundity) and hatched (fertility) were subjected to one-way ANOVA with a *t*-test (*p* < 0.05) using SPSS 19 (IBM Inc, Chicago, IL, USA). The results were plotted using the GraphPad Prism 5 (PRISM 5.0, GraphPad Software, CA, USA) software. A detailed description of the data analysis is given in Supplementary Materials.

## 3. Results

### 3.1. Effect of X-ray Irradiation on the Lifespan of Male Adults of C. pomonella

**Figure 2:**
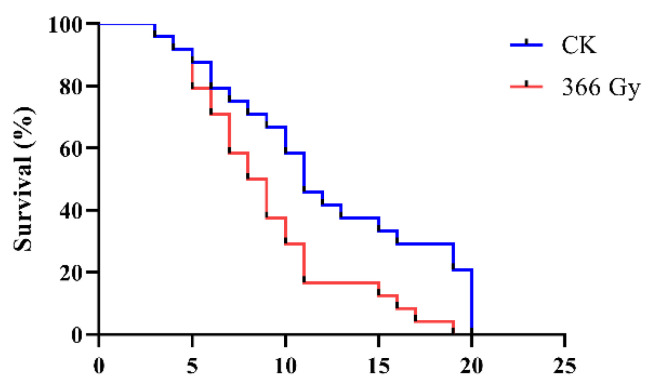
Survival curves of *C. pomonella* irradiated with 366 Gy X-ray irradiation in the field. Three replicates, each with eight males per group, were coupled with eight virgin females. Survival period data for each treatment were recorded and analyzed by means of a *t*-test (*p* < 0.001).

In order to determine whether X-ray irradiation could influence the lifespan of male adults of *C. pomonella*, the between-subjects effects (treatments and days post-treatments) were analyzed using two-way ANOVA (Table S1). Compared with the control group, the irradiation of pupae with 366 Gy of X-ray significantly reduced the life span of *C. pomonella* (F = 74.05, *df* = 1, *p* < 0.001), with average lifespans of 12.29 ± 1.18 d (mean of triplicates ± SE) for the male adults in the control group and 9.13 ± 0.88 d for those in the irradiated group, respectively (Figure 2).

### 3.2. Effect of X-ray Irradiation on the Fecundity of C. pomonella

**Figure 3:**
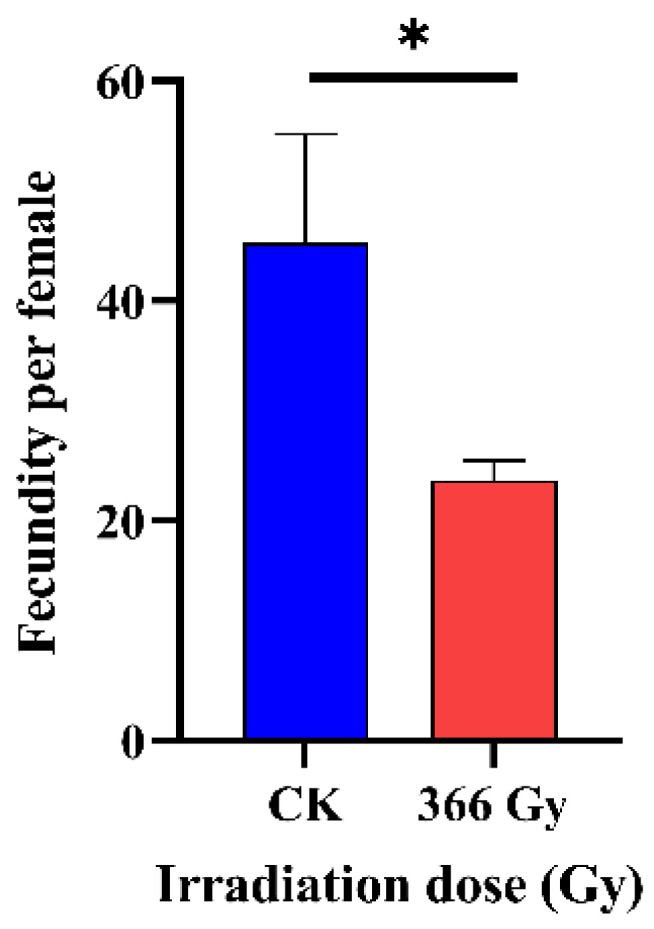
Effect of 366 Gy X-ray irradiation on the fecundity of the *C. pomonella.* Male pupae from 1 day before emergence (8-day-old) were irradiated with 0 Gy (CK) or 366 Gy X-ray, and mated with non-irradiated virgin females, respectively. Three replicates, each with eight males per treatment, were coupled with eight virgin females. The results are shown as the mean ± SD. Error bars represent the standard errors calculated from three replicates. Asterisk (*) on the error bars indicate significant differences analyzed by means of *t*-test (*p* < 0.05).

The females mated with the males irradiated with 366 Gy of X-ray irradiation (23.68 ± 1.80) laid a significantly less (F = 7.86; *df* = 2,6, *p* = 0.021) total number of eggs (fecundity) compared to females mated with the non-irradiated control (45.25 ± 9.92) (Figure 3).

### 3.3. Effect of X-ray Irradiation on the Fertility of C. pomonella

**Figure 4:**
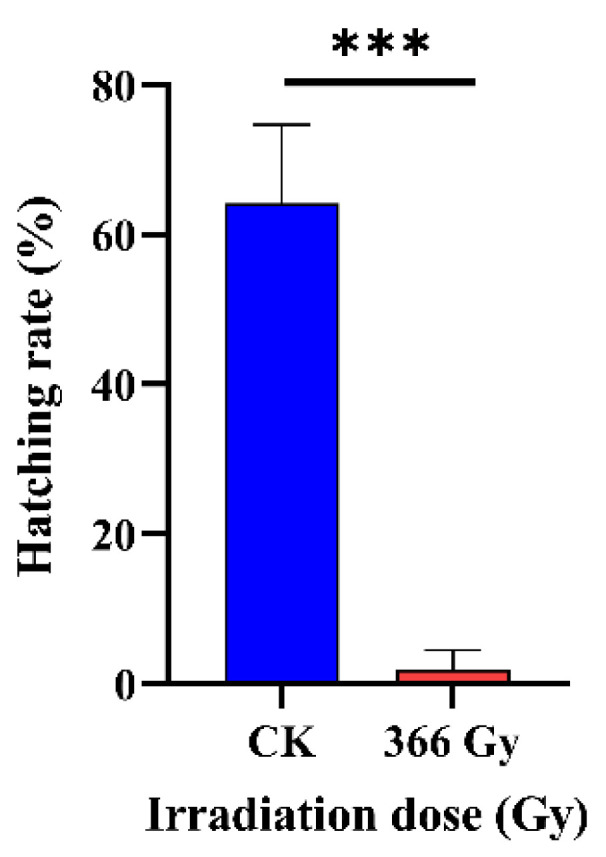
Effect of 366 Gy X-ray irradiation on hatching rate of *C. pomonella* in the orchard. Male pupae from 1 day before emergence (8-day-old) were irradiated with 0 Gy (CK) or 366 Gy, and the eclosed adults were crossed with non-irradiated females. Three replicates, each with eight males per group, were coupled with eight virgin females. The results are shown as the mean ± SD. Error bars represent the standard errors calculated from three replicates. Asterisk (***) on the error bars indicate significant differences analyzed by means of *t*-test (*p <* 0.001).

The hatching rate of eggs (fertility) was significantly influenced by X-ray irradiation in the field, with a hatching rate of 1.91 ± 2.59% for the females mated with the males irradiated with 366 Gy X-ray irradiation, which was significantly lower (F = 89.375, *df* = 2,6, *p* < 0.001) than the rate for the females mated with the non-irradiated males (64.15 ± 10.45%) (Figure 4).

### 3.4. Effect of X-ray Irradiation on the Male Mating Competitiveness of C. pomonella

The egg hatching rate of IM:NM:NF at a ratio of 1:0:1 was 1.91 ± 2.59%, which was significantly (F = 89.375, *df* = 2,6, *p* < 0.001) lower than the ratio of 0:1:1 (egg hatching rate of 64.15 ± 10.45%) and 1:1:1 (egg hatching rate of 63.49 ± 3.57%), with an expected hatching value (E) of 33.03% when male adults were mixed with different matching ratios and paired with normal female adults. The competitive mating index (C) is 0.0088, indicating that 366 Gy of irradiation reduced the mating competitiveness of *C. pomonella* male moths (Table 1).

### 3.5. Effect of Pilot Release of Sterile Male Adults of C. pomonella on Field Fruit Infestation Rate

**Figure A1:**
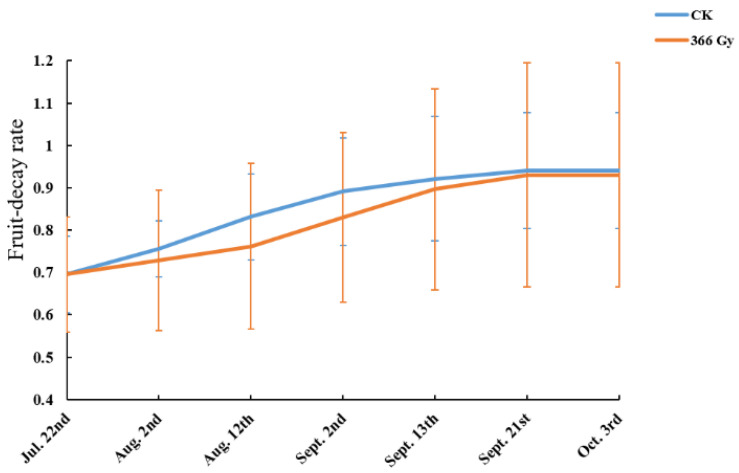
The effect of pilot release of sterile male adults of *C. pomonella* on the fruit infestation rate of Nanguo pear. Male pupae from 1 day before emergence (8-day-old) were irradiated with 366 Gy X-ray, and the emerged adults were released. The fruit infestation rate of orchards with releasing of sterile males was recorded every 10 days post-release to evaluate whether there was a control effect. The results are shown as the mean ± SD. Error bars represent the standard errors calculated from five replicates. An independent sample *t*-test was used to analyze the data (*p* = 0.586).

In order to determine whether the pilot release of sterile male adults of *C. pomonella* could influence the fruit infestation rate of the Nanguo pear, the between-subjects effects (treatments and days post-treatments) were analyzed using two-way ANOVA (Table S2). The results showed that the fruit infestation rate of the orchard with a pilot release of sterile male adults of *C. pomonella* was 0.82 ± 0.37%, and the value in the control group was 0.85 ± 0.37%. There was no significant difference in the fruit infestation rate between the orchard with the released sterile males and the control area during a field investigation from 22 July to 3 October (F = 0.011, *p* > 0.05) (Figure A1; Table S2).

## 4. Discussion

Laboratory results are not fully representative of the fecundity, hatching rate, survival, and mating competitiveness of *C. pomonella* in the field [32,33]. Therefore, we explored the adaptability of the sterile codling moth under natural conditions in a pear orchard. According to our previous studies, the appropriate radiation dose for *C. pomonella* should be ≤366 Gy, and the sterile male insects irradiated by 366 Gy can effectively reduce the mean egg-hatching rate of a target population and lead to its suppression. Our results are in line with the results reported by other researchers, who found that the fertility of the codling moth was close to zero when it was irradiated with 400 Gy by a ^60^Co source [34]. However, the suitable irradiation dose used for South African and Canadian populations was 150 Gy, which is not a dose for complete infertility [35]. These results indicate that in the SIT plan for codling moth control, the radiation dose chosen has gone from 350–400 Gy for sterility to a lower dose for sub-sterility, whereas some scholars still insist on choosing a dose above 300 Gy [23]. Similarly, the results of the field cage experiment in this study show that after mating with the males irradiated with 366 Gy X-ray irradiation, the hatching rate of the females was significantly less than that of the control group; this would cause population depression via sterility. At the same time, mating competitiveness has also decreased. Indeed, an optimal radiation dose employed should raise the levels of sterility induced in the insects, reduce their damage-producing potential without sterility, and compromise their competitiveness in mating with wild moths [36]. The above results indicated that sterile males irradiated with 366 Gy X-ray might be at a disadvantage in competition with wild males. The number of eggs laid by the non-irradiated females after mating with the irradiated males did not decrease significantly, but the mating competitiveness dramatically decreased. These results indicate that when sterile males are released into orchards with more complex environmental conditions than in the laboratory, their competitiveness will be even lower in competing with wild males.

The life span of irradiated males is a crucial parameter in evaluating the quality of sterile insects [37]. A higher irradiation dose may also affect the somatic cells of insects, resulting in a shortened life span [38]. To increase their chance of mating with females, sterile males need to live a long life in the wild [39]. If the life span of sterile males is shortened, the frequency with which they are released and the number of sterile males (quantity) released should be increased to ensure that there are enough of them to compete effectively with wild males. In this study, we released sterile males twice, with 1250 insects each time, due to the life span of sterile males being significantly shortened owing to their being irradiated with 366 Gy X-ray irradiation. We also found a significant reduction in the mating competitiveness of sterile males. The competitiveness of insects comes from their phenotypes, which are determined by their conditions and surrounding environmental conditions. The physiological environments experienced by insects in the laboratory and the field are completely different, and environmental differences, such as feeding conditions, light stimulation, temperature, food composition, or pH, can directly or indirectly affect the phenotypes of insects [40]. This may lead to a further decline in the mating competitiveness of male insects in the field compared with those kept indoors.

Many experiments and projects have proved the feasibility of using SIT technology to control and eliminate pests [40,41,42]. Moreover, the economic benefits of a SIT program can far exceed the cost of suppression or eradication, which has been confirmed in many cases [43]. In the sterile insect release (SIR) program carried out in Canada in 1992, the population density of the codling moth was suppressed by releasing sterile moths [40]. Carpenter and Cross (1993) found that the increase in the number of wild *Helicoverpa zea* was significantly delayed after the release of irradiated male adults by the marker-recovery method in the field, indicating that releasing sterile males is an effective way to control wild populations [41]. However, our results showed that there was an insignificant decrease in the fruit infestation rate between the released sterile males and the control groups. The reason for this result may be that the number of sterile male adults released is insufficient to suppress the number of wild populations in the field. It may also be caused by the decrease in male flying ability and mating competitiveness [42]. We need further study to improve the quality of irradiated males and understand the mechanism of how this dose of X-ray causes *C. pomonella* infertility.

The phenomenon of inherited sterility and its connection with radiation-induced chromosome aberrations has been known since 1935 [44]. Ionizing energy breaks chemical bonds within DNA and other molecules, thereby disrupting normal cellular function in the insect. It is well known that the response of insects to irradiation varies with the species and life stage, as well as the absorbed dose received by the insect [45]. Generally, insects in Lepidoptera are radioresistant due to the holokinetic structure of their chromosomes [46]. Therefore, higher radiation doses are usually required to cause sterility. However, the competitiveness of sterile insects irradiated with high doses in the field is weak [47]. Although irradiation may break the chromosomal structure, fragments and functional genes are not completely lost due to the presence of the filament plates of lepidopteran insects, and these can be passed on to the next generation through germ cells [46,47,48,49]. Thus, research on the radiation sterility of lepidopteran insects has mainly focused on the sub-sterilization dose because such a dose is more advantageous than using complete sterility to control pests, as the F1 generation of irradiated males usually bequeath sterility genes to their offspring [50,51,52]. The release test of the sterile codling moth in British Columbia in 1970 showed that the percentage of apples damaged at harvest after the release of sub-sterile moths irradiated with 25 krad was better than that observed after the release of completely sterile moths irradiated with 40 krad, because they had higher mating competitiveness [53]. Therefore, Lepidoptera do not need to be irradiated with a dose producing 100% sterility [46]. Therefore, releasing adults that are not completely sterile can not only reduce the number of pests in their generation but can also reduce the number of their offspring. A lower irradiation dose can not only reduce the cost of the procedure but can also better realize the continuous control of the number of pests. When the density of the wild population is low, for example, in the overwintering area, genetic sterility technology can be used. Thus, further research is needed to find an incomplete dose more suitable for *C. pomonella*, and a balance between the appropriate sterility and the mating competitiveness of the male is critical to obtain a better control efficacy.

In addition to choosing the appropriate irradiation dose, the release ratio of sterile males is also critical [54]. A proper release ratio can reduce the amount of released sterile male insects, thus saving cost and also making the released sterile male insects more fully utilized [50,54]. Jiang [50] found that the sterility rate of offspring reached 74% when the ratio of irradiated males to non-irradiated males was 12:1 in the mating competitiveness experiment of *Spodoptera frugiperda*. When releasing sterile insects in the field, it may be necessary to have an appropriate release ratio to better control pests. The appropriate proportion for field release may be larger than that measured in the laboratory. For example, in the field experiment of *Anastrepha fraterculus,* the control effect is ideal when the ratio of sterile male insects to wild adult insects reaches 50:1. When the release ratio of sterile males to wild males is 100:1, the sterility rate reaches 70% [54,55]. At present, we are exploring how to determine the proportion of sterile male insects released according to the number of wild pests. A previous study has shown that the recapture rate of *C. pomonella* released on the ground was much lower than that of *C. pomonella* released in the crown, which indicated that different release methods might directly affect the diffusion effect of the sterile moth and that the method of release in high places was more effective. The study also found that the negative impact of releasing the sterile moth in spring would be greater than that of releasing it in autumn [56]. Continuous release for many years also plays an important role in the prevention and control of *C. pomonella*. Following the plan, initiated in August 1992, of producing and releasing the sterile codling moth in Osoyus, Okanagan Canyon, the number of fruit trees damaged by the codling moth was successfully reduced in 1997, which showed that releasing sterile moths was an effective method and could successfully reduce the fruit infestation rate in orchards [57,58]. In the current study, the release of sterile male insects has no significant effect on the fruit infestation rate, which may be associated with the loss of mating competitiveness in irradiated males. Adding active ingredients, such as probiotic microorganisms, into their diets is a potential measure to improve the mating competitiveness of sterile males [59].

## Figures and Tables

**Table 1:** Effect of X-ray irradiation on the mating competitiveness of *C. pomonella* adults in the field.

Matching Ratio (IM:NM:NF) ^1^	Total Egg Production	Number of Eggs Laid/Female	Hatching Rate %		Competitive Capacity (C)
			Observed Value	Expected Value (E)	
0:1:1	300.33 ± 147.70 ab	45.25 ± 9.92 ab	64.15 ± 10.45 a	33.03	0.0088
1:0:1	102.33 ± 12.01 b	23.68 ± 1.80 b	1.91 ± 2.59 b		
1:1:1	534 ± 165.76 a	66.75 ± 20.72 a	63.49 ± 3.57 a		

^1^ Irradiated males (IM), non-irradiated males (NM), and non-irradiated females (NF) were introduced into the cage in the ratios of 0:1:1, 1:0:1, and 1:1:1, respectively, for mating. Three replicates were assessed, each with 0 (at the ratio of 0:1:1) or 8 (at the ratio of 1:0:1 or 1:1:1) IM. The data in the table are the mean ± standard deviation (SD). Letters behind indicate significant differences analyzed by the two-way analysis of variance (ANOVA) using Duncan’s test (*p* < 0.05).

## Data Availability

The data presented in this study are available on request from the corresponding author. The data are not publicly available due to privacy restrictions.

## Supplementary Materials

The following supporting information can be downloaded at: https://www.mdpi.com/article/10.3390/insects14070615/s1. Table S1: Variance analysis of factors influencing the lifespan of male adults of *C. pomonella* (between-subjects effects); Table S2: Variance analysis of factors influencing the fruit infestation rate of Nanguo pear caused by *C. pomonella* (between-subjects effects).
