# Correction: Zhang et al. The Effect of X-ray Irradiation on the Fitness and Field Adaptability of the Codling Moth: An Orchard Study in Northeast China. *Insects* 2023, *14*, 615

**DOI:** 10.3390/insects16090955

**Published:** 2025-09-11

**Authors:** Jinghan Zhang, Shengwang Huang, Shici Zhao, Xingya Wang, Xianming Yang, Huiyuan Zhao, Ping Gao, Yuting Li, Xueqing Yang

**Affiliations:** 1 College of Plant Protection, Shenyang Agricultural University, Shenyang 110866, Chinayutingli2017@syau.edu.cn (Y.L.); 2 Key Laboratory of Economical and Applied Entomology of Liaoning Province, Shenyang 110866, China; 3 State Key Laboratory for Biology of Plant Diseases and Insect Pests, Institute of Plant Protection, Chinese Academy of Agricultural Sciences, Beijing 100193, China; zqbxming@163.com; 4 Hebi Jiaduoke Industry and Trade Co., Ltd., Hebi 458030, China

## Missing Details in Materials and Methods

In the original publication [1], details were missing from the Methods (Section 2.2).

The 8-day-old male pupae were irradiated with 366 Gy X-ray at a dose rate of 12.7083 mGy/s, and their fitness was assessed in a Nanguo pear orchard.

The authors state that the scientific conclusions are unaffected. This correction has been approved by the Academic Editor. The original publication has also been updated.
