# Supplementary material for: The Effect of X-ray Irradiation on the Fitness and Field Adaptability of the Codling Moth: An Orchard Study in Northeast China
Source: Insects. 2023 Jul 7;14(7):615. doi: 10.3390/insects14070615 (PMC10380233; doi:10.3390/insects14070615)
Supplement: Supplementary file 1 [file insects-14-00615-s001.zip › Supplementary Materials.pdf]

Supplementary Materials

Effect of X-ray irradiation on the fitness and field adaptability of the codling moth: an orchard study in northeast China

Jing-Han Zhang [1,2,†], Sheng-Wang Huang [1,2, †], Shi-Ci Zhao [1,2], Xing-Ya Wang [1,2], Xian-Ming Yang [3], Hui-Yuan Zhao [4], Ping Gao [1,2, *], Yu-Ting Li [1,2], Xue-Qing Yang[1,2, *]

[1]College of Plant Protection, Shenyang Agricultural University, Shenyang 110866, Liaoning, China;

[2]Key Laboratory of Economical and Applied Entomology of Liaoning Province, Shenyang 110866, Liaoning, China;

[3] State Key Laboratory for Biology of Plant Diseases and Insect Pests, Institute of Plant Protection, Chinese Academy of Agricultural Sciences, Beijing 100193, China

[4] Hebi Jiaduoke Industry and Trade Co., Ltd, Hebi 458030, Henan Province, China

[†]These authors contributed equally to this work.

[*]Author for correspondence: gaoping1202@163.com(Ping Gao); sling233@hotmail.com (Xue-Qing Yang)

Supplementary Materials and Methods

Effect of X-ray irradiation on the lifespan of male adults of *C. pomonella*

The experiment was repeated in three groups, each with 8 pairs of male and female insects in spawning cages. The experimental male pupae were irradiated with 366 Gy X-ray, and the control group was not irradiated. The survival of each male adult was observed every day. Adults were considered dead when no physical response was detected using an ink brush tool, and dead adults were removed from the cage every day. The lifespan of male adults was recorded.

Significant differences in the mean value of the lifespan were subjected to two-way ANOVA ($P<0.05$) using SPSS 19 (IBM Inc, Chicago, Illinois). Results were plotted using the GraphPad Prism 5 (GraphPad Software, CA) software. Data are presented as the mean of triplicates ± standard deviation (SD).

Effect of X-ray irradiation on the fecundity of *C. pomonella*

The emerging moths from Irradiated and unirradiated male pupae were paired with unirradiated females to allow them to lay eggs on waxed papers. Three groups, each with 8 pairs, were repeated in spawning cages. Waxed papers were collected, replaced, and the number of eggs laid was recorded daily. The mate capsule of the dead female adults was dissected under a stereoscope with 1 × objective (TS-63X, Shanghai Shangguang New Optical Technology Co., Ltd., Shanghai, China) to check whether there is a spermatophore to calculate the single female oviposition. A female adult was considered contains successfully mated with a spermatophore. The fecundity of *C. pomonella* was calculated based on the mating number and total fecundity of each group of female insects.

Significant differences in the mean value of the fecundity were subjected to *t*-test analysis using SPSS 19 (IBM Inc, Chicago, Illinois). Results were plotted using the GraphPad Prism 5 (GraphPad Software, CA) software. Data are presented as the mean of triplicates ± standard deviation (SD).

Effect of X-ray irradiation on the fertility of *C. pomonella*

The emerging moths from Irradiated and unirradiated male pupae were paired with unirradiated females to allow them to lay eggs on waxed papers. Three groups, each with 8 pairs, were repeated in spawning cages. Waxed papers were collected, replaced, and the number of eggs laid and larvae hatched in the experimental group and the control group was counted daily. The percentage of the number of total larvae hatched/eggs laid was used to calculate the fertility of *C. pomonella*

Significant differences in the mean value of the lifespan were subjected to *t*-test analysis using SPSS 19 (IBM Inc, Chicago, Illinois). Results were plotted using the GraphPad Prism 5 (GraphPad Software, CA) software. Data are presented as the mean of triplicates ± standard deviation (SD).

Effect of X-ray irradiation on the male mating competitiveness of *C. pomonella*

Irradiated males (IM), non-irradiated males (NM), and non-irradiated females (NF) were introduced into waxed paper spawning cages at the ratio of 0: 1: 1, 1: 0: 1, and 1: 1: 1, respectively for mating. Then, the total number of eggs laid and hatched in each group was recorded. Each mating combination was repeated three times, and each repeat contained 8 mating combinations. Total egg production, Number of eggs laid, and Observed value were subjected to one-way analysis of variance (ANOVA)

using SPSS 19 (IBM Inc., Chicago, IL). Significant differences were analyzed by Duncan's test.

Effect of pilot-releasing of sterile male adults of *C. pomonella* on fruit infestation rate on Nanguo pear

A total of 20 fruit trees were randomly selected, and a five-point sampling method was used to investigate the fruit-infestation rate in the four corners (east, south, west, and north) of pear trees in control and the experimental orchards, respectively. The fruits with hanging insect droppings and wormholes were counted as rotten fruits, and the number was used to calculate the fruit infestation rate of the Nanguo pear. The fruit infestation rate of each group was checked every 10 days post-release to evaluate whether there was a control effect between two orchards.

Significant differences in the mean value of the fruit infection rate of experimental and control orchards before and after releasing sterile male insects were subjected to two-way ANOVA (P<0.05) using SPSS 19 (IBM Inc, Chicago, Illinois). Results were plotted using the GraphPad Prism 5 (GraphPad Software, CA) software.

Table S1. Variance analysis of factors influencing the lifespan of male adults of *C. pomonella* (between-subjects effects).

Source	*df*	*p*	F
Treatments	19	< 0.001	64.16
Days post treatments	1	< 0.001	74.05
Days post treatments * Treatments	19	< 0.001	1.77

Table S2. Variance analysis of factors influencing the fruit infestation rate of Nanguo pear caused by *C. pomonella* (between-subjects effects).

Source	*df*	*p*	F
Treatments	1	0.756	0.097
Monitoring date	6	0.715	0.62
Treatments * Monitoring date	6	1	0.011
